# From cartilage–sensory nerve crosstalk to clinically actionable pain phenotyping in osteoarthritis

**DOI:** 10.1016/j.jot.2026.101169

**Published:** 2026-07-02

**Authors:** Zhewei Ma, Yiquan Ji

**Affiliations:** Department of Orthopedics, The Second Hospital of Jiaxing, China

Dear Editor,

We read with great interest the review by Meng et al. [1], which provides a timely and comprehensive synthesis of cartilage-sensory nerve crosstalk in osteoarthritis (OA) progression. By moving beyond a structure-centred view of OA, the authors highlight how cartilage-derived inflammatory mediators, nerve growth factor (NGF), neuropeptides, and mechanosensitive ion channels may jointly contribute to persistent pain and disease progression. We believe that this mechanistic framework has considerable translational value. However, three issues deserve further emphasis before these insights can be converted into clinically actionable strategies.

First, cartilage-sensory nerve crosstalk should not be considered a molecular axis in isolation but rather be part of a multidimensional pain-phenotyping approach. OA pain is not limited to the severity of radiographic findings or cartilage damage; it includes peripheral nociceptive input, neuroinflammation, peripheral and central sensitization, psychological factors, activity status, other diseases, patient expectations, etc [[2], [3], [4]]. Therefore, future studies might include magnetic resonance imaging (MRI) measures of synovitis or bone marrow lesions (BMLs) along with quantitative sensory testing (QST), neuropathic-like symptom assessment, patient-reported outcomes, and functional measures, to help differentiate inflammatory, mechanical, neurogenic, centrally sensitised, or mixed pain phenotypes. Based on the neurobiological focus of the cartilage-sensory nerve hypothesis, this framework can be further enhanced by adding assessments of neural structure and function, such as temporal summation, conditioned pain modulation, neuropathic pain questionnaires, circulating or synovial neuroinflammatory mediators, and emerging neuroimaging methods that study peripheral and central sensitization [3,5]. This distinction is crucial for patient stratification to specific treatments and for minimizing the well-documented mismatch between structural severity of OA and patient symptoms.

Second, there is a need to better model the whole-joint neuroimmune and biomechanical microenvironment. The hypothesis reviewed is about cartilage-sensory neuron connections, but OA involves the whole joint. Synovium and subchondral bone influence nociceptive signalling and tissue damage under conditions of infrapatellar fat pad, vascular architecture, immune cells and load. Reductionist chondrocyte-neuron models are still employed to investigate particular biological pathways and determine the causes and effects of them experimentally. However, such models should be regarded as complementary to all-encompassing systems rather than replacing them. Humanised co-culture systems, explant culture models of dorsal root ganglia, and joint-on-a-chip platforms that incorporate synoviocytes, immune cells, osteochondral units, adipose tissue, cyclic mechanical loading and sensory neurons may be particularly useful for studying clinically relevant amplifiers of OA pain [6,7]. In addition to neurite outgrowth and neurone excitability, these models should assess the degradation of the extracellular matrix, synovial inflammatory activity, neuropeptide release and mechanically triggered nociceptive responses. This would render preclinical systems more relevant to clinical pain phenotype and therapeutic screening.

Third, the goal of therapeutic translation should be to keep the difference between symptomatic analgesia and disease modification.Blocking NGF, calcitonin gene-related peptide, transient receptor potential channels, PIEZO channels, or other neuroinflammatory molecules could result in a reduction of nociceptive signals without compromising structural protection. The clinical experience with treatment with anti-NGFs has indicated that strong pain relief should be weighed against joint safety, including rapidly progressive osteoarthritis and other serious joint-related adverse events [8]. At the same time, the good analgesia that can be achieved by neural-targeting strategies has also helped to provide clinical support for the application of sensory-neural pathways in OA pain. Recent Phase 2 data for LEVI-04, a p75 neurotrophin receptor fusion protein that targets neurotrophin signalling, have also shown that optimising neural-targeted therapies may improve pain and function under a favourable safety profile [9]. Future studies of the cartilage-sensory nerve hypothesis should therefore not be limited to short-term pain relief. This should include measures of pain intensity, pain during movement, physical function, use of rescue analgesics, imaging progression, soluble biomarkers and adjudicated joint safety events. Stratified trial designs could be of special interest, with patient selection based on baseline neural sensitization, inflammatory activity, mechanically overloaded joints, or biomarker-defined neuroimmune activation.

To conclude, Meng et al. have supplied a significant mechanistic framework to comprehend OA pain beyond only cartilage loss. The next step is to move from this biology to clinically useful phenotypes, physiologically accurate models, and trial endpoints that distinguish pain suppression from disease modification. A Cartilage-Neuroimmune-Mechanical Phenotyping Pipeline Can Help Connect Molecular Discovery and personalised OA treatment.

## **Declaration of competing interest**

The authors declare that they have no known competing financial interests or personal relationships that could have appeared to influence the work reported in this manuscript.
